# *spatialMaxent*: Adapting species distribution modeling to spatial data

**DOI:** 10.1002/ece3.10635

**Published:** 2023-10-24

**Authors:** Lisa Bald, Jannis Gottwald, Dirk Zeuss

**Affiliations:** ^1^ Department of Geography, Environmental Informatics Philipps‐University Marburg Marburg Germany

**Keywords:** Maxent, model tuning, NCEAS dataset, open‐source software, spatial validation, species distribution modeling

## Abstract

Conventional practices in species distribution modeling lack predictive power when the spatial structure of data is not taken into account. However, choosing a modeling approach that accounts for overfitting during model training can improve predictive performance on spatially separated test data, leading to more reliable models. This study introduces *spatialMaxent* (https://github.com/envima/spatialMaxent), a software that combines state‐of‐the‐art spatial modeling techniques with the popular species distribution modeling software Maxent. It includes forward‐variable‐selection, forward‐feature‐selection, and regularization‐multiplier tuning based on spatial cross‐validation, which enables addressing overfitting during model training by considering the impact of spatial dependency in the training data. We assessed the performance of *spatialMaxent* using the National Center for Ecological Analysis and Synthesis dataset, which contains over 200 anonymized species across six regions worldwide. Our results show that *spatialMaxent* outperforms both conventional Maxent and models optimized according to literature recommendations without using a spatial tuning strategy in 80 percent of the cases. *spatialMaxent* is user‐friendly and easily accessible to researchers, government authorities, and conservation practitioners. Therefore, it has the potential to play an important role in addressing pressing challenges of biodiversity conservation.

## Graphical abstract

*spatialMaxent* outperforms classical and tuned species distribution modeling with Maxent by accounting for overfitting during model training and tuning. *spatialMaxent* is a standalone open‐source Java software that is accessible through the command line and via a graphical user interface for easy access and broad applicability.

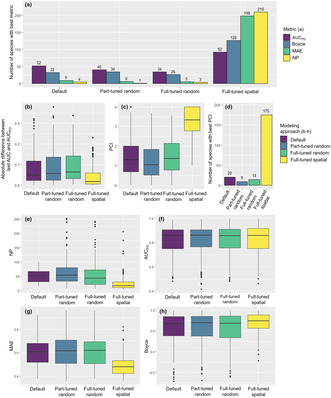

## 1 INTRODUCTION

To decelerate the human‐induced loss of biodiversity, 140 countries recently agreed on protecting one third of the planet's lands, coastal areas, and inland waters by the end of the decade (COP15, 2022). In order to achieve a significant impact on species protection, it is essential to identify areas of high conservation value. Species distribution modeling (SDM) and habitat suitability models (herein both types are referred to as SDMs) have become an indispensable tool in ecological research and nature conservation (Villero et al., 2017). These models have the potential to forecast the distribution of invasive or endangered species under climate change scenarios and to identify areas of high value for the protection of endangered species (Porfirio et al., 2014). Further, government authorities are increasingly relying on these techniques as a basis for conservation management decisions (Guisan et al., 2013; Sofaer et al., 2019; Villero et al., 2017). However, the results of SDMs cannot be fully relied upon due to their often inadequate performance on spatially separated test data (i.e., data not used to train the model and spatially separated from the data used for model training; Lee‐Yaw et al., 2022), especially if they are tuned with spatially dependent data.

One reason for poor SDM performance is the insufficient or even complete lack of model tuning (i.e., finding the best set of model parameters). A review by Feng et al. (2019) found that only 45% of studies using SDMs in 2017 and 2018 reported essential SDM model parameters necessary for reproducibility. Among the various SDM approaches available, the open‐source software Maxent (Phillips et al., 2006, 2017) is among the most popular (Guillera‐Arroita et al., 2015) because it is readily available via a user‐friendly graphical user interface (GUI; Merow et al., 2013; Morales et al., 2017). The complexity and performance of Maxent models are essentially determined by two model parameters: (1) the regularization‐multiplier (RM), which is a numerical value that controls the complexity of the models; and (2) feature classes, which are a series of mathematical transformations of the variables for modeling complex relationships (e.g., linear, hinge; see: Merow et al., 2013). Phillips and Dudík (2008) identified default settings for these parameters by modeling 225 species from six regions worldwide contained in the National Center for Ecological Analysis and Synthesis (NCEAS) dataset (Elith et al., 2020; Phillips & Dudík, 2008). The assumption that these default parameters are a replacement for model tuning is outdated because several studies have shown that better performances can be achieved with parameters that are specifically determined for each species (Bao et al., 2022; Hallgren et al., 2019; Radosavljevic & Anderson, 2014). However, for this popular software, most studies (~97%) demonstrated that little effort was made to tune models beyond the default settings provided in Maxent (Morales et al., 2017).

Another concerning reason for poor SDM performance is the disregard of overfitting during model training and validation (e.g. during cross‐validation runs; Ploton et al., 2020; Schratz et al., 2019). The scientific community has been aware that spatial proximity also implies greater similarity and thus non‐independence of data points since the formulation of Tobler's first law of geography (Tobler, 1970). However, the common practice for evaluating the results of SDMs is still to randomly exclude 10–20% of the target species locations from model training in order to subsequently use them for model testing (Sillero & Barbosa, 2021). Several studies have demonstrated that training and validation with spatially dependent data often leads to inflated performance metrics, overly complex models, and a poor performance on spatially separated test data (Kattenborn et al., 2022; Meyer et al., 2018, 2019; Ploton et al., 2020; Roberts et al., 2017; Valavi et al., 2019). For instance, variable selection algorithms that select predictors based on cross‐validation with spatially separated folds (e.g., spatial‐block cross‐validation; Valavi et al., 2019) can decrease overfitting and increase the predictive performance of models (Le Rest et al., 2014; Meyer et al., 2018).

There are numerous R packages available for utilizing Maxent, including “dismo” (Hijmans et al., 2022), “SDMtune” (Vignali et al., 2020), or “ENMeval” (Kass et al., 2021; Muscarella et al., 2014), to name just a few. For a comprehensive overview of R packages for SDM, refer to the review by Sillero et al. (2023). Among them, the R package “ENMeval” has gained great popularity due to its easy provision of automatic tuning and spatial cross‐validation for Maxent models. This has already made the application of Maxent models much easier for users. However, there is currently no software that combines spatial validation and tuning together with an automatic variable selection, which should lead to a significant improvement in modeling (Meyer et al., 2018; Zeng et al., 2016).

In this study, we implemented functionalities to reduce overfitting in a Maxent advancement (called “*spatialMaxent*”) with the same GUI as the original Maxent software. In particular, we implemented forward‐variable‐selection (FVS) and forward‐feature‐selection (FFS) algorithms together with regularization‐multiplier tuning based on spatial cross‐validation to reduce overfitting during model tuning. We assessed the performance of *spatialMaxent* in terms of model complexity and performance with the NCEAS dataset across six regions of the world by repartitioning the occurrence records of 218 species of this dataset into spatial blocks (Valavi et al., 2019). We calculated four different model evaluation metrics on spatially separated test data and compared our results to models based on Maxent's default settings and tuned models in which spatial dependence was not considered during model training. We demonstrate that *spatialMaxent* improves predictive performance in 80% of cases and clearly outperforms classical as well as tuned species distribution modeling with Maxent.

## 2 SPATIALMAXENT

A possible explanation for the lack of tuning in published studies using Maxent is attributed to an easily accessible GUI, which facilitates the broad applicability of the software but without providing ready‐to‐use tuning options (Morales et al., 2017). To overcome this limitation, we developed a Maxent advancement, “*spatialMaxent* 1.0.0,” which encompasses a spatial validation and tuning method, a variable selection procedure, feature selection, and regularization‐multiplier tuning.

Recent studies have demonstrated that accounting for spatial dependence at the model tuning stage results in better performing and less overfitted models (Meyer et al., 2018). The selection of the best model parameter configuration ultimately depends on the learning success of the model, which in turn is determined by the validation strategy. Hence, in spatial modeling, spatial validation is not just a validation strategy but is an essential tuning strategy. Tuning while accounting for the spatial structure of the data accounts for each possible model parameter configuration being validated on data that is as independent from the training data as possible. This allows strict exclusion of parameters that do not contribute to an improved model performance on spatially separated data. In the context of SDMs, the selected model parameters are forced toward parameters such as selected variables that best reflect the habitat of a species.

**FIGURE 1:**
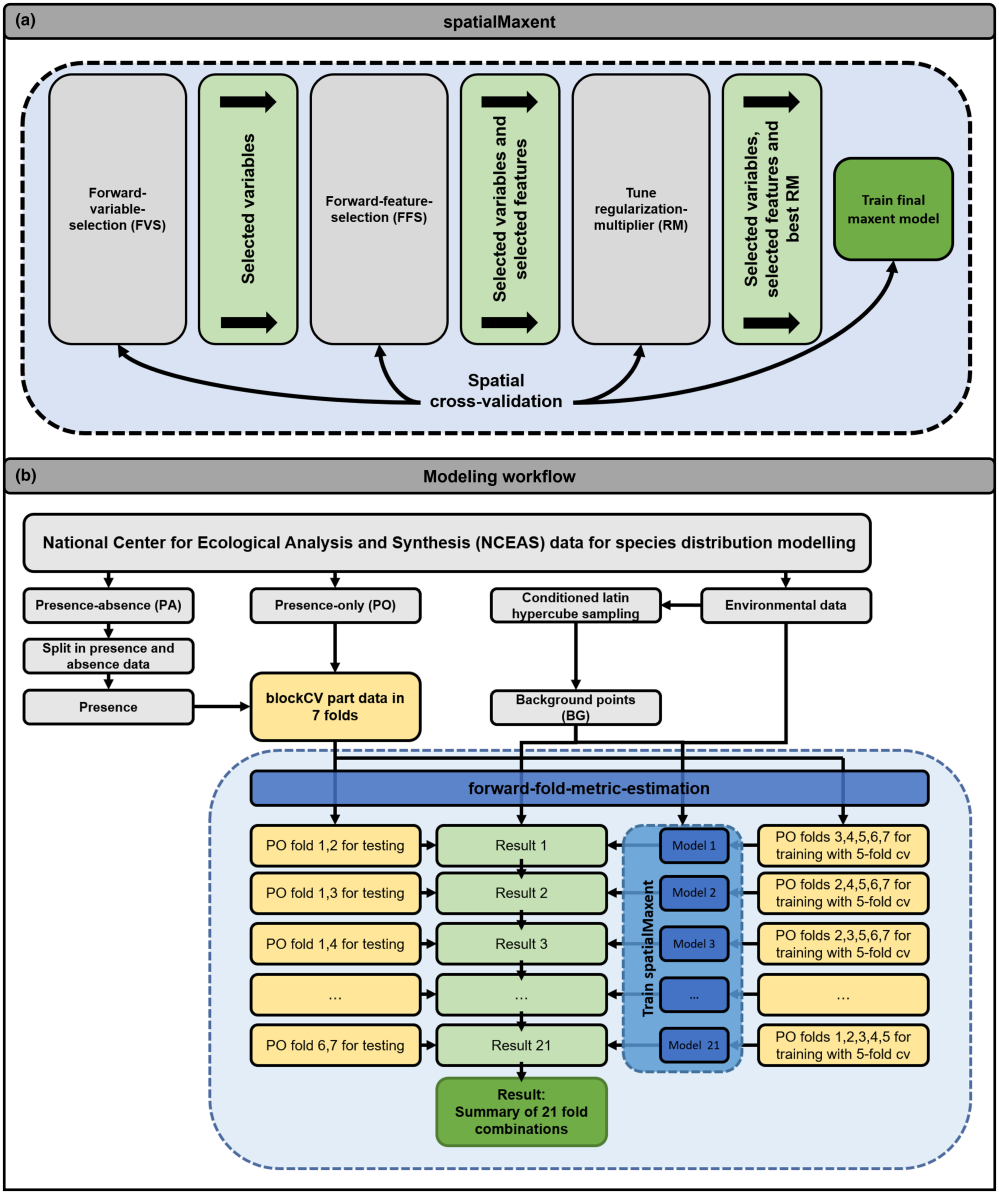
Modeling workflow of *spatialMaxent* (a) Software structure of *spatialMaxent*. A total of three tuning algorithms are executed successively. First, the best variables are selected by FVS and spatial cross‐validation. Next, the best combination of mathematical transformations of the selected variables (Maxent feature class; hinge, linear, etc.) are selected by FFS and spatial cross‐validation. Finally, the best regularization‐multiplier (RM) is selected based on spatial cross‐validation. After the determination of the optimal parameters (variables, feature classes, RM), the model is validated by n‐fold spatial cross‐validation and results for each fold are reported in the Maxent results file and the familiar results html. (b) Data preparation and forward‐fold‐metric‐estimation (FFME). Presence records of the NCEAS data were grouped into seven spatial folds. Five spatial folds were used for model cross‐validation. The other two folds were held back for testing on spatially separated data. This was repeated for all possible combinations of training and test folds, thus, a total of 21 iterations. For each of the 21 models, four evaluation metrics were calculated. The median value of each metric was calculated.

*spatialMaxent* uses three consecutive tuning processes in model training (Figure 1a): (1) FVS; (2) FFS; and (3) regularization‐multiplier (RM) tuning.

All tuning steps in *spatialMaxent* can be performed with random cross‐validation or spatial cross‐validation. *spatialMaxent* should be applied with FVS, FFS, and regularization‐multiplier tuning, and all models should be validated with spatial cross‐validation. However, it is also possible to only use parts of the tuning procedure.

The implementation of *spatialMaxent* was performed in Java using openjdk 18. It also runs on Java SE 18 and newer versions. *spatialMaxent* 1.0.0. is based on Maxent version 3.4.4. It is available as a stand‐alone .*jar* file and can be used in the same way as the original Maxent either via the GUI or the command line. *spatialMaxent* is distributed under the MIT license. Documentation, a tutorial, and the source code are hosted on GitHub (https://github.com/envima/spatialMaxent).

### 2.1 Spatial cross‐validation

*spatialMaxent* implements a spatial cross‐validation as the internal validation method to account for overfitting during the three tuning steps. The presence points must be externally grouped by spatially clustered locations (clusters, blocks) beforehand by using for instance blocking methods as implemented in the “blockCV” R package (Valavi et al., 2019). In each cross‐validation iteration, one of the blocks is held back as validation datum while the models are trained with data from the remaining blocks (Meyer et al., 2018; Valavi et al., 2019). Next, an n‐fold cross‐validation is performed, where the number of replicates/folds is equal to the number of distinct blocks.

### 2.2 Forward‐variable‐selection

To perform FVS, models are first trained with all possible combinations of two variables. The best combination is selected by *spatialMaxent* based on either test‐gain or test area under the curve (AUC). The decision parameter determining the best model is averaged over the results of all folds. The best performing two‐variable combination is trained together with all remaining variables separately and the best model is selected again. This step is repeated until no further improvement is obtained by adding more variables to the model (for more details on FVS see: Meyer et al., 2018). All subsequent models are computed using only the variables selected by FVS.

Pseudocode for FVS (Meyer et al., 2018):
**for** *each resampling iteration* **do**
 train models using all possible 2‐variable combinations and calculate model performance with spatial cross‐validation
**end**
Keep the best 2‐variable model (model_best_)
**for** *each additional number of variables i, i = 3 …N* **do**
 **for** each remaining variable V_R_ **do**
 **for** each resampling iteration **do**
 train models using the variables of model best and V_R_ and calculate model performance with spatial cross‐validation
 **end**
 **end**
 **if** mean (error of model i) > mean(error of model_best_) **then**
 *Break*
 **end**
Keep the best performing i‐variable model (model_best_)
**end**



### 2.3 Forward‐feature‐selection

Feature classes or features are a series of mathematical transformations of the covariates for modeling complex relationships. The FFS follows the same basic concept as FVS, except that the first models are trained with only one of the feature classes. The model with the best feature class is selected and another feature is added until no improvement in model performance is observed. The subsequent models are trained with only the selected variables and features.

Pseudocode for FFS (Meyer et al., 2018):
**for** *each resampling iteration* **do**
 train models using all possible features and calculate model performance with spatial cross‐validation
**end**
Keep the best feature model (model_best_)
**for** *each additional number of features i, i = 2 …N* **do**
 **for** each remaining feature F_R_ **do**
 **for** each resampling iteration **do.**
 train models using the features of model_best_ and F_R_ and calculate model performance with spatial cross‐validation
 **end**
 **end**
 **if** mean (error of model i) > mean(error of model_best_) **then**
 *Break*
 **end**
Keep the best performing i‐feature model (model_best_)
**end**



### 2.4 Regularization‐multiplier tuning

The RM is a numerical value that controls the complexity of the models. RM tuning is completed by computing models with RMs from RM_min_ to RM_max_ in RM_increase_ increments.

Pseudocode for RM tuning:
**for** *each resampling iteration* **do**
 train models using all possible regularization‐multipliers RM_min_ to RM_max_ in RM_increase_ increments and calculate model  performance with spatial cross‐validation
**end**
Keep the best regularization‐multiplier model (model_best_)



The final model is trained with the selected variables, selected features, best RM, and all presence points. This procedure is extremely computationally intensive which means that large quantities of variables are linked with large computational costs. To reduce computation time for these extensive tuning schemes, the FVS is fully parallelized in *spatialMaxent*. Nevertheless, depending on the computing capacity and dataset, the procedure can take several hours or even days on standard computers.

## 3 MATERIALS AND METHODS

All pre‐ and post‐processing of the data and evaluation of the models (sections 3.2 and 3.3) were performed in R version 4.2.1 (R Core Team, 2022).

A tutorial explaining all work‐steps using Canada from the NCEAS dataset as an example is provided online (https://envima.github.io/spatialMaxent/).

### 3.1 Modeling

We compared four modeling approaches to assess the performance of *spatialMaxent* in terms of predictive ability and model complexity: (1) Maxent model trained with five‐fold random cross‐validation and the default settings (*default model*) of Maxent; (2) Maxent model trained with FFS, RM tuning, and a five‐fold random cross‐validation *(part‐tuned random model*); (3) Maxent model trained with FVS, FFS, RM tuning, and five‐fold spatial cross‐validation (*full‐tuned spatial model*), representing the full functionalities of *spatialMaxent*; and (4) Maxent was trained with FFS, FVS, RM tuning, and random five‐fold cross‐validation (*full‐tuned random model*). The *default* and *part‐tuned random* Maxent models are inspired by modeling approaches employed by Valavi et al. (2022). Modeling approach four allowed demonstration of the importance of spatial validation and that good results are only obtained by variable and feature selection when each model is trained with a spatial validation approach.

The models containing RM tuning were tuned from RM_min_ = 0.5 to RM_max_ = 7 in steps of 0.5. We used the test AUC as the parameter for selecting the best model.

### 3.2 Data preparation

The default parameters provided in Maxent were determined by modeling 225 species in a total of six regions worldwide (Phillips & Dudík, 2008). The NCEAS dataset has recently been published as an open benchmark dataset explicitly assembled for comparing SDM methods (Elith et al., 2020; data available from Open Science Framework (OSF): https://osf.io/kwc4v/).

The NCEAS dataset covers six regions: Australian wet tropics, Ontario Canada, New South Wales Australia, New Zealand, South American countries, and Switzerland. The species themselves are anonymized and only assigned to a biological group. The data consists of presence‐only (PO) records, presence‐absence (PA) records, background points (BP), and environmental predictors as raster layers for each species (spatial resolution between 80 m and 1 km). The PO and BP data are intended to train and validate the SDM models, and the PA data to test them. For a more detailed description of the NCEAS dataset, see (Elith et al., 2020).

#### 3.2.1 Presence‐only and presence‐absence data

**FIGURE 2:**
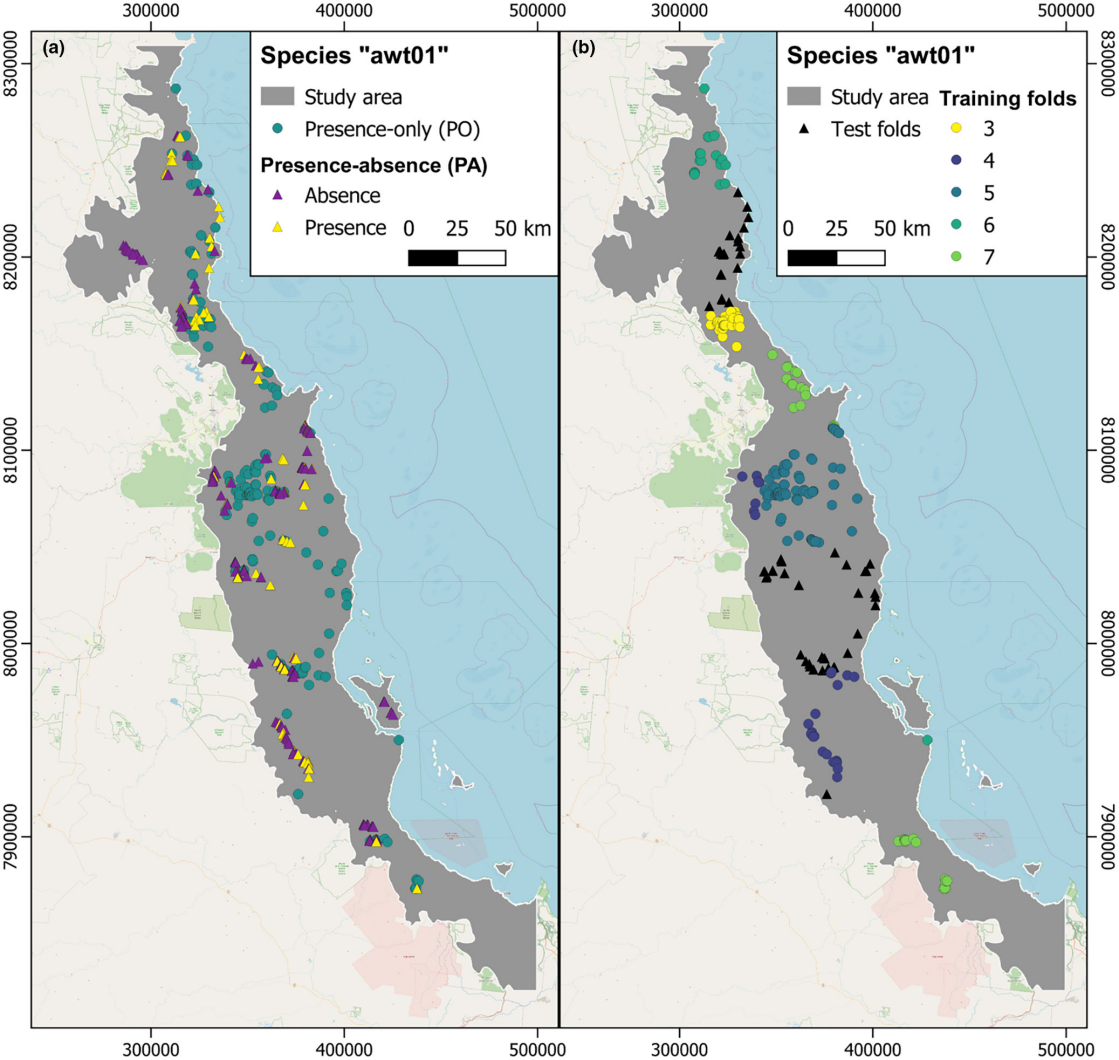
Example species “awt01” in the Australian wet tropics from the National Center for Ecological Analysis and Synthesis (NCEAS) dataset. (a) Presence‐only (PO) and Presence‐absence (PA) data. (b) Presence‐only points from the PO and PA data as seen in (a), parted into seven spatial folds with the R package “blockCV.” In the first of 21 FFME rounds for the performance evaluation, the black triangles were used for independent testing without being part of the modeling. The points of the folds 3–7 were used for model parameterization based on spatial cross‐validation. Data: Elith et al. (2020) and OpenStreetMap (2023).

The PA data is provided as a separate dataset independent from PO data with the intention to use the former for model testing. Notably, the presence points in both datasets exhibit a pattern similar to a random separation of training and test data (Figure 2), and no spatial delineation between training and test data is visible. To enable spatial cross‐validation and evaluation with a reasonable number of records per species, we combined the presence records from the PO and the PA data to one new dataset which was subsequently divided into spatial blocks using the “blockCV” R package (Valavi et al., 2019).

From the combined presence points, we only selected species with at least 35 occurrence records because we aimed for five‐fold cross‐validation and evaluation on two external folds. Thus, we created seven cross‐validation folds with at least five data records each, leaving a total of 218 out of 225 species for modeling. Next, we partitioned the data into spatially distinct blocks (spatial folds) for spatial cross‐validation. These spatial folds were created with the function spatialBlock() from the R package “blockCV” (version 2.1.4; Valavi et al., 2019). The function spatialBlock() divides the study region into spatial blocks of squared shape and distributes these blocks across a user‐defined number of folds. We repeated this process 200 times for each species and the fold assignment with the most balanced number of presence records per species over all folds was chosen for further modeling (Valavi et al., 2019).

#### 3.2.2 Background points

Elith et al. (2020) stated that the 10,000 randomly distributed background points across each region in the original NCEAS dataset, which is the default number in the Maxent software, might be insufficient for some of the regions. Consequently, previous studies used 50,000 randomly distributed background points for each region (Valavi et al., 2022). The issue of optimal sampling size and distribution of background points remains a major challenge in SDM which will not be discussed in this present study. As our study focuses on a comparison between modeling approaches and not on calculating context‐specific ecologically meaningful SDMs, we argue that a comparison is justified as long as modeling conditions are held constant between different approaches. Thus, we also used 10,000 background points (default setting and recommended by Merow et al., 2013) for each region in the NCEAS dataset but did not sample them randomly over the entire study area. Instead, we used conditioned Latin hypercube sampling (Minasny & McBratney, 2006) as implemented in the R package “clhs” (version 0.9.0; Roudier, 2011) to distribute the background points over the study area whereby all variables of the environmental data were represented as well as possible. Background points within the same pixel as the environmental layers as presence records were removed.

#### 3.2.3 Selection of folds and forward‐fold‐metric‐estimation

Out of the seven spatial folds of presence records, five were used for model training and two were used as spatially separated test data. One of the most crucial aspects in spatial validation is the spatial distribution of training, validation, and test points because the selection of the folds being removed from the data for testing can result in large differences in determining model quality. For instance, a model might predict one fold perfectly even though it has never been part of the training data, but might fail for others. Randomly excluding one or more spatial folds for external evaluation therefore provides an incomplete picture of the model quality. To obtain a comprehensive picture of which modeling approach performs best on the NCEAS dataset, we proposed to remove the effect of random selection of spatial folds for calculating model quality by using a forward‐fold‐metric‐estimation (FFME). In FFME, models are calculated for all possible combinations of training and test data and each model is evaluated with its respective spatially separated test data. The median of all result metrics is then used to assess the overall quality of the modeling approach (Figure 1b). Consequently, every PO point will eventually be part of the model training, while simultaneously the models are always evaluated with spatially independent data.

### 3.3 Evaluation

We utilized four different evaluation criteria for assessing which modeling approach performs best. We first used the AUC and then the mean absolute error (MAE) as proposed by Konowalik and Nosol (2021). Both metrics were calculated for each FFME‐run on spatially separated test data using the R package “Metrics” (version 0.1.4; Hamner & Frasco, 2018). The MAE is defined as the average absolute deviance between the predicted value (= 1) and the observed value ([0,1]) at presence points. To calculate AUC, we randomly sampled the same number of background points as available presence points. As the AUC is not initially intended to be calculated on background points but on absence data, we follow the suggestion of Yackulic et al. (2013) and will from here on refer to the AUC as AUC presence‐only (AUC_PO_) to establish a clear distinction between AUC values calculated on PA and PO data. We are aware of the general problems associated with these metrics, especially the AUC (Lobo et al., 2008) and thus used these only to compare between modeling approaches and not to make statements of absolute model performance. As a third metric, we calculated the Boyce‐Index (Boyce et al., 2002) with the R package “ecospat” (version 3.3; Di Cola et al., 2017) using the prediction raster and spatially separated test data. Finally, as a fourth metric we used the number of parameters of each model as an indicator of model complexity.

These four metrics were determined for each fold of the FFME and their median value was calculated for each species separately. The assessment of which modeling approach was the best *for each species* was made based on the highest Boyce‐Index, highest AUC_PO_, lowest MAE, and least complex model (i.e., model with the minimum number of parameters). We compared the metric values of each species and assigned the species to the approach with the best value for further comparison. The best modeling approach *for each metric* (exemplary calculation in Table 1) was then defined as the one with the highest number of assigned species.

**TABLE 1:** Exemplary determination of the best modeling approach for AUC_PO_ values.

Species	Modeling approach				Best AUC_PO_
	Full‐tuned spatial	Default	Full‐tuned random	Part‐tuned random	
Awt01	**0.5623669**	0.5026700	0.4984056	0.4559949	Full‐tuned spatial
Awt02	0.5013007	**0.6569951**	0.6133472	0.6052319	Default
Awt03	**0.6455855**	0.5647337	0.5976606	0.5957396	Full‐tuned spatial
Swi30	0.8891490	**0.8914484**	0.8900276	0.8897441	Default

*Note*: Median values for each modeling approach over all forward‐fold‐metric‐estimation folds. The result of the best modeling approach is indicated in bold.

To express the overall performance of each modeling approach in conjunction with model complexity, we created a single performance‐complexity‐index (PCI) based on all four metrics. To do this, we scaled the metrics of all models for each species from 0 to 1 with inverted scales of MAE and the number of parameters. The sum of all four scaled metrics per species and modeling approach formed the PCI (exemplary calculation Table 2).

**TABLE 2:** Exemplary calculation of the Performance‐Complexity‐Index (PCI) for the species “awt01.”

Modeling approach	Full‐tuned spatial	Default	Full‐tuned random	Part‐tuned random
Boyce	0.299	−0.138	−0.295	−0.326
Boyce scaled	1	0.3008	0.0496	0
AUC_PO_	0.5623669	0.50267	0.4984056	0.4559949
AUC_PO_ scaled	1	0.438791223	0.398701726	0
Number parameters	16	57	74	86
Number parameters scaled	1	0.414285714	0.171428571	0
MAE	0.485469422	0.765030005	0.80018211	0.826386672
MAE scaled	1	0.17997525	0.076864876	0
PCI	4	1.333852187	0.696595173	0

*Note*: For each metric (AUC_PO_, MAE, Boyce, and number of parameters), the values for all four modeling approaches are scaled from 0 to 1 with inverted scales of MAE and the number of parameters. The sum of all four scaled metrics per species and modeling approach formed the PCI.

## 4 RESULTS

**FIGURE 3:**
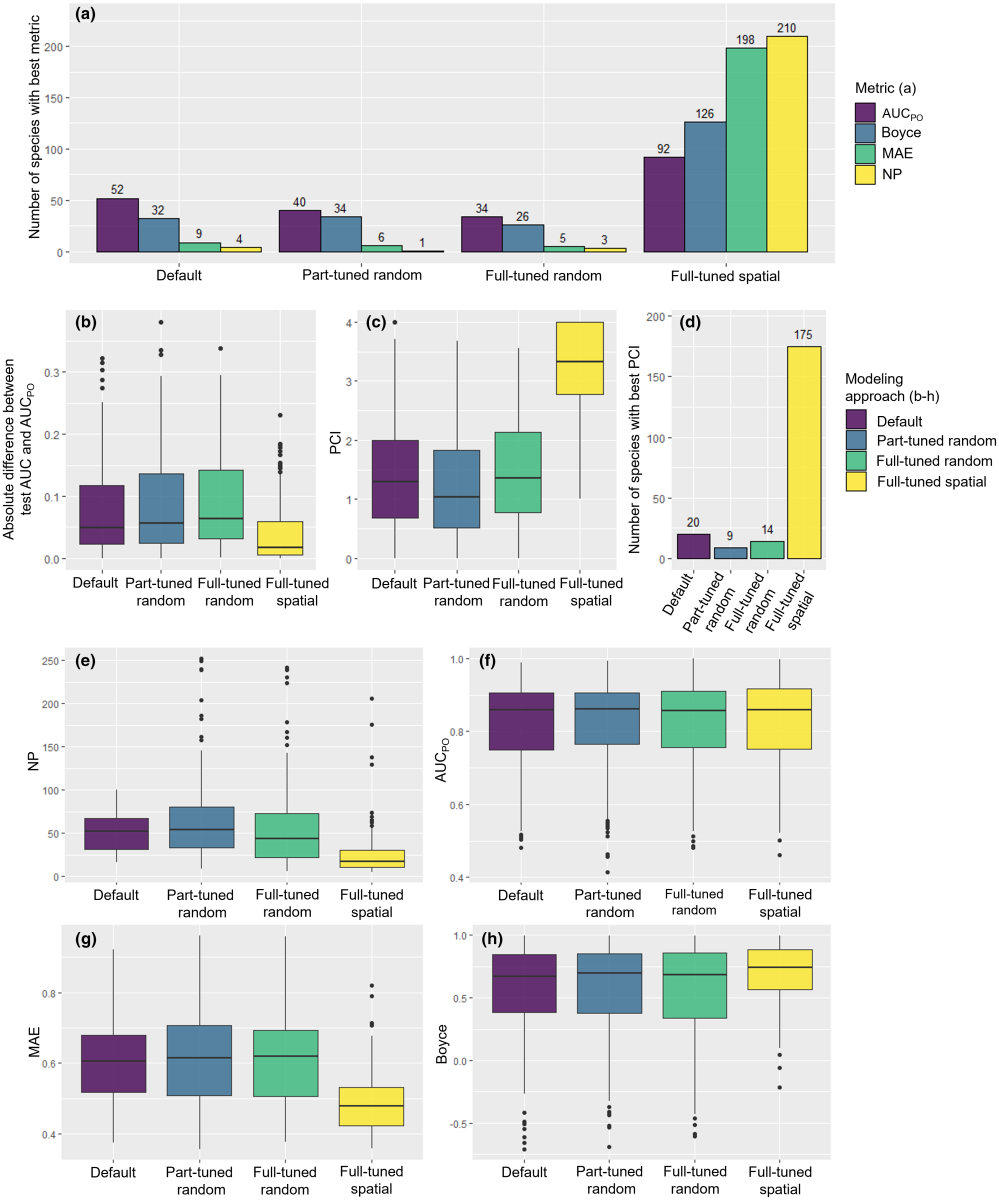
Comparison of the performance of modeling approaches based on species. (a) Number of species in each modeling approach with the best area‐under‐the‐curve value (AUC_PO_), Boyce‐Index (Boyce), Mean Absolute Error (MAE), and number of parameters (NP). Note that the number of species per metric across the four modeling approaches sums up to 218, which is to the total number of species used. (b) Absolute difference between the test AUC, as given by Maxent and AUC_PO_, calculated using spatially separated test data. (c) Boxplots of the Performance‐Complexity‐Index calculated from scaled AUC_PO_, MAE, Boyce‐Index, and number parameters for 218 species. (d) Number of species with the best Performance‐Complexity‐Index, as calculated from the scaled AUC_PO_, MAE, Boyce‐Index, and number parameters. (e) Number of parameters (NP) for all 218 species by modeling approach. (f) AUC_PO_ for all 218 species by modeling approach. (g) MAE for all 218 species by modeling approach. (h) Boyce‐Index for all 218 species by modeling approach.

**FIGURE 4:**
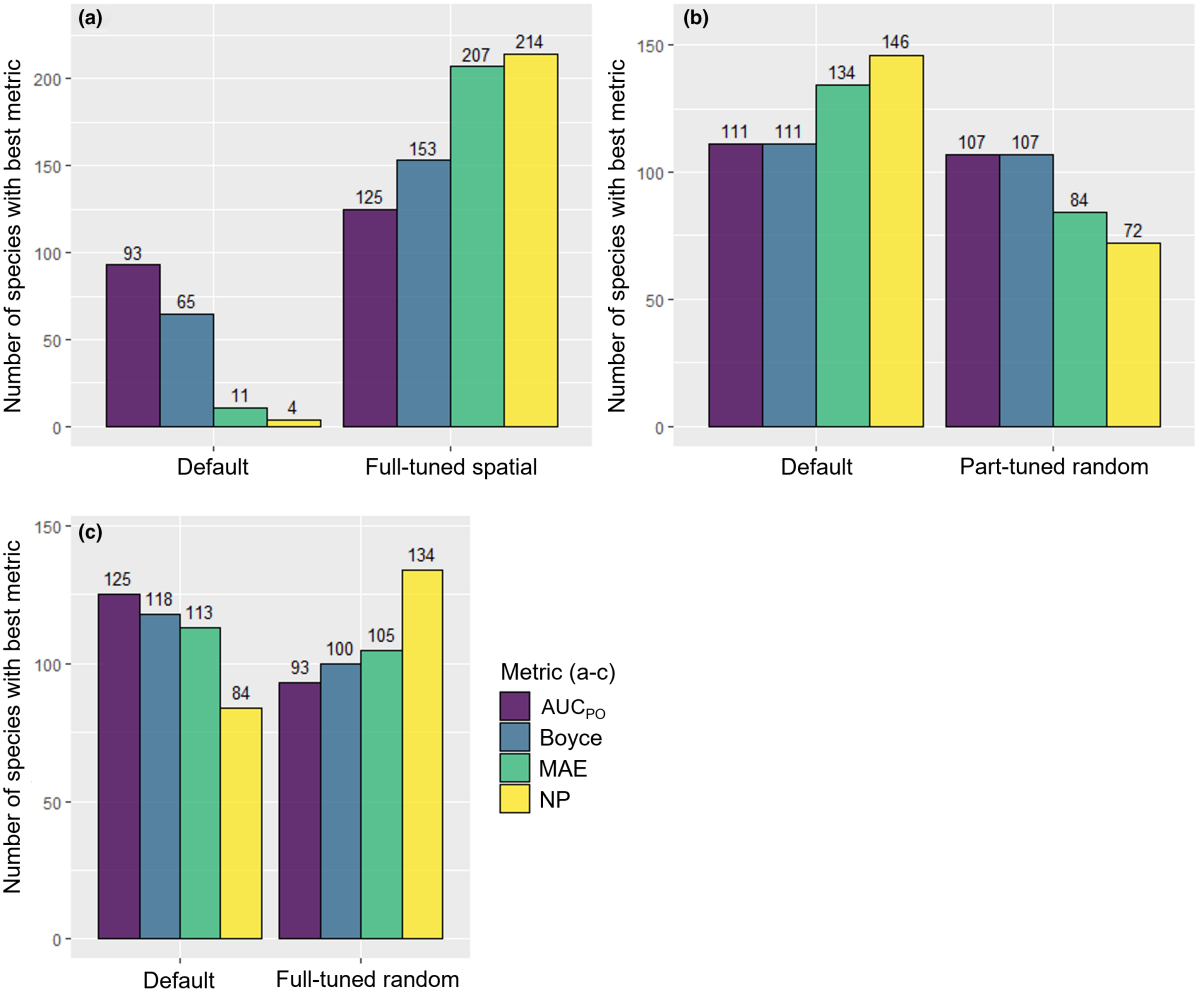
Number of species out of 218 (*y*‐axis) which reached the maximum Boyce‐Index, the maximum AUC_PO_ value, the minimum MAE, or the minimum number of parameters for (a) the full‐tuned spatial modeling approach compared to the default modeling approach, (b) the full‐tuned random modeling approach compared to the default modeling approach, and (c) the part‐tuned random modeling approach compared to the default modeling approach.

The *full‐tuned spatial* modeling approach achieved the best results compared to the other three approaches for all four metrics. The least complex models for 210 out of 218 species were produced. The results were worst for the AUC_PO_ values, where for a total of 92 of 218 species the best AUC_PO_ value was achieved. The next best modeling approach was the *default* modeling approach, achieving the best results for 52 of 218 species. However, the AUC_PO_ values calculated for the four methods had a very similar range; therefore, it was difficult to determine which modeling approach provided the best results (Figure 3f). The Boyce‐Index, MAE, and number of parameters clearly demonstrated that the *full‐tuned spatial* modeling approach performed better on spatially separated test data (Figure 3e,g,h). The ratio of the *full‐tuned spatial* modeling approach to the next best modeling approach for the Boyce‐Index and the MAE was 126 to 34 and 198 to 9, respectively (Figure 3a). In general, the *default* modeling approach was the next best compared to the *full‐tuned spatial* modeling approach; however, the three modeling approaches using random cross‐validation (*default, part‐tuned random, full‐tuned random*) recorded a similar range. A direct comparison of each modeling approach to the *default* modeling approach can be found in Figure 4.

The results for the PCI calculated from scaled Boyce‐Index, number of parameters, MAE, and AUC_PO_ for all species for each modeling approach can be seen in Figure 3c,d. The *full‐tuned spatial* modeling approach was best for >80% of the species when directly compared with the other modeling approaches (Figure 3d). The other three modeling approaches exhibited very similar results.

The AUC_PO_ values of the validation on spatially separated test data were significantly closer to the test AUC of the cross‐validation of *spatialMaxent* compared to the other three modeling approaches (Figure 3b). *spatialMaxent* also allowed a more realistic assessment of the quality of the models based on internal model error rather than the models derived with random cross‐validation.

As mentioned earlier, the performance improvement of *spatialMaxent* is accompanied by higher computational costs. Among the four modeling approaches used, the median computational time on one thread was 2.07 min for the default modeling approach, 4.32 min for the part‐tuned random modeling approach, and 16.2 min each for the full‐tuned spatial and full‐tuned random modeling approaches. Since the FVS can be executed in parallel, the processing time for the last two approaches can be improved. When run on 10 threads, the median processing time was 9.33 min for the full‐tuned random approach and 3.39 min for the full‐tuned spatial approach. Therefore, *spatialMaxent*'s computational demands, while higher for the full‐tuned settings, remain manageable for researchers using standard hardware configurations.

## 5 DISCUSSION

Ignoring spatial dependence in data and a lack of tuning are among the most common mistakes in SDM (Sillero & Barbosa, 2021). The software Maxent in its standard version offers the user a simple way to perform SDM. However, the current Maxent GUI lacks automatic tuning options and functionalities to account for overfitting, which might be the reason for missing model tuning attempts. In our study, we consolidated current knowledge regarding best practices in spatial modeling and incorporated them into *spatialMaxent*, an advancement of the popular Maxent software.

Here, we demonstrated that appropriate tuning and variable‐selection methods that account for overfitting result in more reliable models with improved predictive performance. However, even when spatial cross‐validation in *spatialMaxent* is used, care must be taken when arranging the spatial folds. For instance, if data is clustered too heavily, spatial cross‐validation is not possible because a sufficient independence between cross‐validation folds cannot be achieved (Meyer & Pebesma, 2022).

Compared to the random cross‐validation results, the internal model AUC of the *spatialMaxent* approach also remained much closer to the AUC_PO_ obtained by external validation with spatially separated test data. This reiterates the spatial cross‐validation during model training better reflecting the predictive power and potential error of the model compared to the overoptimistic results obtained via random cross‐validation.

SDM is increasingly being used in a wide variety of ecological fields, and offers a broad range of applications in decision‐making (Araújo et al., 2019). Therefore, the importance of improving the quality of SDMs is essential. Given the popularity of Maxent and its current application in conservation research and practice, our results based on the NCEAS dataset are concerning. Even if model tuning without spatial cross‐validation is applied, results are often not better than the default settings. Only the combination of model tuning with spatial cross‐validation resulted in better performance and less complex models. An analysis of the impact of fine‐tuning on the response curves is also possible in *spatialMaxent*, as it provides the same response curve output as Maxent. However, due to the anonymization of species in this study, we refrained from doing so. Future research can explore the influence of fine‐tuning in *spatialMaxent* on response curves using non‐anonymized species data for deeper insights.

Most previous studies which reviewed the use of Maxent default settings in SDM applications only examined peer‐reviewed academic publications. However, the application of Maxent as a conservation planning tool by government authorities is common and offers great practical application in nature conservation. Thus, there is a high probability that the lack of adequate tuning and proper evaluation is even more pronounced in that sector. The resulting models are still treated as realistic and used as a tool for managing endangered species, biodiversity conservation, natural resource management, and studying the impact of climate change (Guillera‐Arroita et al., 2015). Direct negative consequences for endangered species may occur if interventions in the environment are performed using results from these default models (Lee‐Yaw et al., 2022). By implementing the tuning processes and spatial validation directly into the popular Maxent GUI, we hope to promote the creation of SDMs that are meaningful beyond the training data and thereby support nature conservation.

*spatialMaxent* is a valuable software for researchers, government authorities, and conservation practitioners. It can be used to identify areas of high conservation value, improve the accuracy of predictions for invasive or endangered species under climate change scenarios, and aid in conservation management decisions. By providing reliable results and being user‐friendly, this software can be an important tool to support the achievement of the recently established Global COP15 (2022) biodiversity conservation targets. However, there is no one‐fits‐all solution for SDM (Qiao et al., 2015), and even if *spatialMaxent* improves SDMs in an easily accessible way, this does not free users from careful quality control.

## AUTHOR CONTRIBUTIONS

**Lisa Bald:** Formal analysis (equal); methodology (equal); software (lead); validation (equal); visualization (equal); writing – original draft (equal); writing – review and editing (equal). **Jannis Gottwald:** Conceptualization (lead); formal analysis (equal); methodology (equal); software (supporting); supervision (equal); validation (equal); visualization (equal); writing – original draft (equal); writing – review and editing (equal). **Dirk Zeuss:** Funding acquisition (lead); project administration (lead); supervision (equal); writing – review and editing (equal).

## CONFLICT OF INTEREST STATEMENT

We have no conflict of interest to declare.

## Data Availability

A ready‐to‐use jar file of *spatialMaxent* can be found at https://github.com/envima/spatialMaxent. *spatialMaxent* is distributed as open‐source software under the MIT License. Release guides, documentation, and the source code are hosted on GitHub (https://github.com/envima/spatialMaxent). *spatialMaxent* requires a Java 18 SE (or newer) environment to run. A tutorial explaining all work‐steps of this study using the National Center for Ecological Analysis and Synthesis (NCEAS) dataset with Canada as an example is provided at https://envima.github.io/spatialMaxent/. The NCEAS dataset by Elith et al. (2020) is used to build the models in this study; it is available at the Open Science Framework (OSF) repository at https://doi.org/10.17605/OSF.IO/KWC4V. *spatialMaxent* is continuously being developed at the Lab of Environmental Informatics at the Phillips‐University of Marburg, Germany. Further development is driven by the requirements of associated projects and open‐source contributors.

## References

[ece310635-bib-0001] Araújo, M. B. , Anderson, R. P. , Márcia Barbosa, A. , Beale, C. M. , Dormann, C. F. , Early, R. , Garcia, R. A. , Guisan, A. , Maiorano, L. , Naimi, B. , O'Hara, R. B. , Zimmermann, N. E. , & Rahbek, C. (2019). Standards for distribution models in biodiversity assessments. Science Advances, 5, eaat4858. 30746437 10.1126/sciadv.aat4858 PMC6357756

[ece310635-bib-0002] Bao, R. , Li, X. , & Zheng, J. (2022). Feature tuning improves MAXENT predictions of the potential distribution of *Pedicularis longiflora* Rudolph and its variant. PeerJ, 10, e13337. 10.7717/peerj.13337 35529480 PMC9074863

[ece310635-bib-0003] Boyce, M. S. , Vernier, P. R. , Nielsen, S. E. , & Schmiegelow, F. K. A. (2002). Evaluating resource selection functions. Ecological Modelling, 157, 281–300. 10.1016/S0304-3800(02)00200-4

[ece310635-bib-0004] COP15 Biodiversity Conference Outcome . (2022). Convention on biological diversity website . https://prod.drupal.www.infra.cbd.int/sites/default/files/2022‐12/221219‐CBD‐PressRelease‐COP15‐Final_0.pdf

[ece310635-bib-0005] Di Cola, V. , Broennimann, O. , Petitpierre, B. , Breiner, F. T. , D'Amen, M. , Randin, C. , Engler, R. , Pottier, J. , Pio, D. , Dubuis, A. , Pellissier, L. , Mateo, R. G. , Hordijk, W. , Salamin, N. , & Guisan, A. (2017). Ecospat: An R package to support spatial analyses and modeling of species niches and distributions. Ecography, 40, 774–787. 10.1111/ecog.02671

[ece310635-bib-0006] Elith, J. , Graham, C. , Valavi, R. , Abegg, M. , Bruce, C. , Ferrier, S. , Ford, A. , Guisan, A. , Hijmans, R. J. , Huettmann, F. , Lohmann, L. , Loiselle, B. , Moritz, C. , Overton, J. , Peterson, A. T. , Phillips, S. , Richardson, K. , Williams, S. , Wiser, S. K. , … Zimmermann, N. E. (2020). Presence‐only and presence‐absence data for comparing species distribution modeling methods. Biodiversity Informatics, 15, 69–80. 10.17161/bi.v15i2.13384

[ece310635-bib-0007] Feng, X. , Park, D. S. , Walker, C. , Peterson, A. T. , Merow, C. , & Papeş, M. (2019). A checklist for maximizing reproducibility of ecological niche models. Nature Ecology & Evolution, 3, 1382–1395. 10.1038/s41559-019-0972-5 31548646

[ece310635-bib-0008] Guillera‐Arroita, G. , Lahoz‐Monfort, J. J. , Elith, J. , Gordon, A. , Kujala, H. , Lentini, P. E. , McCarthy, M. A. , Tingley, R. , & Wintle, B. A. (2015). Is my species distribution model fit for purpose? Matching data and models to applications: Matching distribution models to applications. Global Ecology and Biogeography, 24, 276–292. 10.1111/geb.12268

[ece310635-bib-0009] Guisan, A. , Tingley, R. , Baumgartner, J. B. , Naujokaitis‐Lewis, I. , Sutcliffe, P. R. , Tulloch, A. I. T. , Regan, T. J. , Brotons, L. , McDonal‐Madden, E. , Mantyka‐Pringle, C. , Martin, T. G. , Rhodes, J. R. , Maggini, R. , Setterfield, S. A. , Elith, J. , Schwartz, M. W. , Wintle, B. A. , Broennimann, O. , Austin, M. , … Buckley, Y. M. (2013). Predicting species distributions for conservation decisions. Ecology Letters, 16, 1424–1435. 10.1111/ele.12189 24134332 PMC4280402

[ece310635-bib-0010] Hallgren, W. , Santana, F. , Low‐Choy, S. , Zhao, Y. , & Mackey, B. (2019). Species distribution models can be highly sensitive to algorithm configuration. Ecological Modelling, 408, 108719. 10.1016/j.ecolmodel.2019.108719

[ece310635-bib-0011] Hamner, B. , & Frasco, M. (2018). Metrics: Evaluation metrics for machine learning . R Package Version 0.1.4.

[ece310635-bib-0012] Hijmans, R. J. , Phillips, S. , Leathwick, J. , & Elith, J. (2022). Dismo: Species distribution modeling . R Package Version 1.3‐9.

[ece310635-bib-0013] Kass, J. M. , Muscarella, R. , Galante, P. J. , Bohl, C. L. , Pinilla‐Buitrago, G. E. , Boria, R. A. , Soley‐Guardia, M. , & Anderson, R. P. (2021). ENMeval 2.0: Redesigned for customizable and reproducible modeling of species' niches and distributions. Methods in Ecology and Evolution, 12(9), 1602–1608. 10.1111/2041-210X.13628

[ece310635-bib-0014] Kattenborn, T. , Schiefer, F. , Frey, J. , Feilhauer, H. , Mahecha, M. D. , & Dormann, C. F. (2022). Spatially autocorrelated training and validation samples inflate performance assessment of convolutional neural networks. ISPRS Open Journal of Photogrammetry and Remote Sensing, 5, 100018. 10.1016/j.ophoto.2022.100018

[ece310635-bib-0015] Konowalik, K. , & Nosol, A. (2021). Evaluation metrics and validation of presence‐only species distribution models based on distributional maps with varying coverage. Scientific Reports, 11, 1482. 10.1038/s41598-020-80062-1 33452285 PMC7811024

[ece310635-bib-0016] Le Rest, K. , Pinaud, D. , Monestiez, P. , Chadoeuf, J. , & Bretagnolle, V. (2014). Spatial leave‐one‐out cross‐validation for variable selection in the presence of spatial autocorrelation: Spatial leave‐one‐out cross‐validation. Global Ecology and Biogeography, 23(7), 811–820. 10.1111/geb.12161

[ece310635-bib-0017] Lee‐Yaw, J. A. , McCune, J. L. , Pironon, S. , & Sheth, S. N. (2022). Species distribution models rarely predict the biology of real populations. Ecography, 2022, e05877. 10.1111/ecog.05877

[ece310635-bib-0018] Lobo, J. M. , Jiménez‐Valverde, A. , & Real, R. (2008). AUC: A misleading measure of the performance of predictive distribution models. Global Ecology and Biogeography, 17, 145–151. 10.1111/j.1466-8238.2007.00358.x

[ece310635-bib-0019] Merow, C. , Smith, M. J. , & Silander, J. A. (2013). A practical guide to MaxEnt for modeling species' distributions: What it does, and why inputs and settings matter. Ecography, 36, 1058–1069. 10.1111/j.1600-0587.2013.07872.x

[ece310635-bib-0020] Meyer, H. , & Pebesma, E. (2022). Machine learning‐based global maps of ecological variables and the challenge of assessing them. Nature Communications, 13, 2208. 10.1038/s41467-022-29838-9 PMC9033849 35459230

[ece310635-bib-0021] Meyer, H. , Reudenbach, C. , Hengl, T. , Katurji, M. , & Nauss, T. (2018). Improving performance of spatio‐temporal machine learning models using forward feature selection and target‐oriented validation. Computational Geosciences, 101, 1–9. 10.1016/j.envsoft.2017.12.001

[ece310635-bib-0022] Meyer, H. , Reudenbach, C. , Wöllauer, S. , & Nauss, T. (2019). Importance of spatial predictor variable selection in machine learning applications – Moving from data reproduction to spatial prediction. Ecological Modelling, 411, 108815. 10.1016/j.ecolmodel.2019.108815

[ece310635-bib-0023] Minasny, B. , & McBratney, A. B. (2006). A conditioned Latin hypercube method for sampling in the presence of ancillary information. Computational Geosciences, 32, 1378–1388. 10.1016/j.cageo.2005.12.009

[ece310635-bib-0024] Morales, N. S. , Fernández, I. C. , & Baca‐González, V. (2017). MaxEnt's parameter configuration and small samples: Are we paying attention to recommendations? A systematic review. PeerJ, 5, e3093. 10.7717/peerj.3093 28316894 PMC5354112

[ece310635-bib-0025] Muscarella, R. , Galante, P. J. , Soley‐Guardia, M. , Boria, R. A. , Kass, J. M. , Uriarte, M. , & Anderson, R. P. (2014). ENMeval: An R package for conducting spatially independent evaluations and estimating optimal model complexity for MAXENT ecological niche models. Methods in Ecology and Evolution, 5(11), 1198–1205. 10.1111/2041-210X.12261

[ece310635-bib-0026] OpenStreetMap . (2023). Base map from OpenStreetMap . https://www.openstreetmap.org/copyright

[ece310635-bib-0027] Phillips, S. J. , Anderson, R. P. , Dudík, M. , Schapire, R. E. , & Blair, M. E. (2017). Opening the black box: An open‐source release of Maxent. Ecography, 40(7), 887–893. 10.1111/ecog.03049

[ece310635-bib-0028] Phillips, S. J. , Anderson, R. P. , & Schapire, R. E. (2006). Maximum entropy modeling of species geographic distributions. Ecological Modelling, 190, 231–259. 10.1016/j.ecolmodel.2005.03.026

[ece310635-bib-0029] Phillips, S. J. , & Dudík, M. (2008). Modeling of species distributions with Maxent: New extensions and a comprehensive evaluation. Ecography, 31, 161–175. 10.1111/j.0906-7590.2008.5203.x

[ece310635-bib-0030] Ploton, P. , Mortier, F. , Réjou‐Méchain, M. , Barbier, N. , Picard, N. , Rossi, V. , Dormann, C. , Cornu, G. , Viennois, G. , Bayol, N. , Lyapustin, A. , Gourlet‐Fleury, S. , & Pélissier, R. (2020). Spatial validation reveals poor predictive performance of large‐scale ecological mapping models. Nature Communications, 11, 4540. 10.1038/s41467-020-18321-y PMC7486894 32917875

[ece310635-bib-0031] Porfirio, L. L. , Harris, R. M. B. , Lefroy, E. C. , Hugh, S. , Gould, S. F. , Lee, G. , Bindoff, N. L. , & Mackey, B. (2014). Improving the use of species distribution models in conservation planning and management under climate change. PLoS One, 9, e113749. 10.1371/journal.pone.0113749 25420020 PMC4242662

[ece310635-bib-0032] Qiao, H. , Soberón, J. , & Peterson, A. T. (2015). No silver bullets in correlative ecological niche modelling: Insights from testing among many potential algorithms for niche estimation. Methods in Ecology and Evolution, 6, 1126–1136. 10.1111/2041-210X.12397

[ece310635-bib-0033] R Core Team . (2022). R: A language and environment for statistical computing. R Foundation for Statistical Computing. https://www.R‐project.org/

[ece310635-bib-0034] Radosavljevic, A. , & Anderson, R. P. (2014). Making better Maxent models of species distributions: Complexity, overfitting and evaluation. Journal of Biogeography, 41, 629–643. 10.1111/jbi.12227

[ece310635-bib-0035] Roberts, D. R. , Bahn, V. , Ciuti, S. , Boyce, M. S. , Elith, J. , Guillera‐Arroita, G. , Hauenstein, S. , Lahoz‐Monfort, J. J. , Schröder, B. , Thuiller, W. , Warton, D. I. , Wintle, B. A. , Hartig, F. , & Dormann, C. F. (2017). Cross‐validation strategies for data with temporal, spatial, hierarchical, or phylogenetic structure. Ecography, 40, 913–929. 10.1111/ecog.02881

[ece310635-bib-0036] Roudier, P. (2011). Clhs: A R package for conditioned Latin hypercube sampling . R package version 0.9.0.

[ece310635-bib-0037] Schratz, P. , Muenchow, J. , Iturritxa, E. , Richter, J. , & Brenning, A. (2019). Hyperparameter tuning and performance assessment of statistical and machine‐learning algorithms using spatial data. Ecological Modelling, 406, 109–120. 10.1016/j.ecolmodel.2019.06.002

[ece310635-bib-0038] Sillero, N. , & Barbosa, A. M. (2021). Common mistakes in ecological niche models. International Journal of Geographical Information Systems, 35, 213–226. 10.1080/13658816.2020.1798968

[ece310635-bib-0039] Sillero, N. , Campos, J. C. , Arenas‐Castro, S. , & Barbosa, A. M. (2023). A curated list of R packages for ecological niche modelling. Ecological Modelling, 476, 110242. 10.1016/j.ecolmodel.2022.110242

[ece310635-bib-0040] Sofaer, H. R. , Jarnevich, C. S. , Pearse, I. S. , Smyth, R. L. , Auer, S. , Cook, G. L. , Edwards, T. C. , Guala, G. F. , Howard, T. G. , Morisette, J. T. , & Hamilton, H. (2019). Development and delivery of species distribution models to inform decision‐making. Bioscience, 69, 544–557. 10.1093/biosci/biz045

[ece310635-bib-0041] Tobler, W. R. (1970). A computer movie simulating urban growth in the Detroit region. Economic Geography, 46, 234. 10.2307/143141

[ece310635-bib-0043] Valavi, R. , Elith, J. , Lahoz‐Monfort, J. J. , & Guillera‐Arroita, G. (2019). blockCV: An r package for generating spatially or environmentally separated folds for k‐fold cross‐validation of species distribution models. Methods in Ecology and Evolution, 10, 225–232. 10.1111/2041-210X.13107

[ece310635-bib-0044] Valavi, R. , Guillera‐Arroita, G. , Lahoz‐Monfort, J. J. , & Elith, J. (2022). Predictive performance of presence‐only species distribution models: A benchmark study with reproducible code. Ecological Monographs, 92, e01486. 10.1002/ecm.1486

[ece310635-bib-0045] Vignali, S. , Barras, A. G. , Arlettaz, R. , & Braunisch, V. (2020). SDMtune: An R package to tune and evaluate species distribution models. Ecology and Evolution, 10(20), 11488–11506. 10.1002/ece3.6786 33144979 PMC7593178

[ece310635-bib-0046] Villero, D. , Pla, M. , Camps, D. , Ruiz‐Olmo, J. , & Brotons, L. (2017). Integrating species distribution modelling into decision‐making to inform conservation actions. Biodiversity and Conservation, 26, 251–271. 10.1007/s10531-016-1243-2

[ece310635-bib-0047] Yackulic, C. B. , Chandler, R. , Zipkin, E. F. , Royle, J. A. , Nichols, J. D. , Campbell Grant, E. H. , & Veran, S. (2013). Presence‐only modelling using MAXENT: When can we trust the inferences? Methods in Ecology and Evolution, 4, 236–243. 10.1111/2041-210x.12004

[ece310635-bib-0048] Zeng, Y. , Low, B. W. , & Yeo, D. C. J. (2016). Novel methods to select environmental variables in MaxEnt: A case study using invasive crayfish. Ecological Modelling, 341, 5–13. 10.1016/j.ecolmodel.2016.09.019

